# Supplementary material for: Lsr2 acts as a cyclic di-GMP receptor that promotes keto-mycolic acid synthesis and biofilm formation in mycobacteria
Source: Nat Commun. 2024 Jan 24;15:695. doi: 10.1038/s41467-024-44774-6 (PMC10808224; doi:10.1038/s41467-024-44774-6)
Supplement: Supplementary file 1 — Supplementary Information [file 41467_2024_44774_MOESM1_ESM.pdf]

Supplemental Material

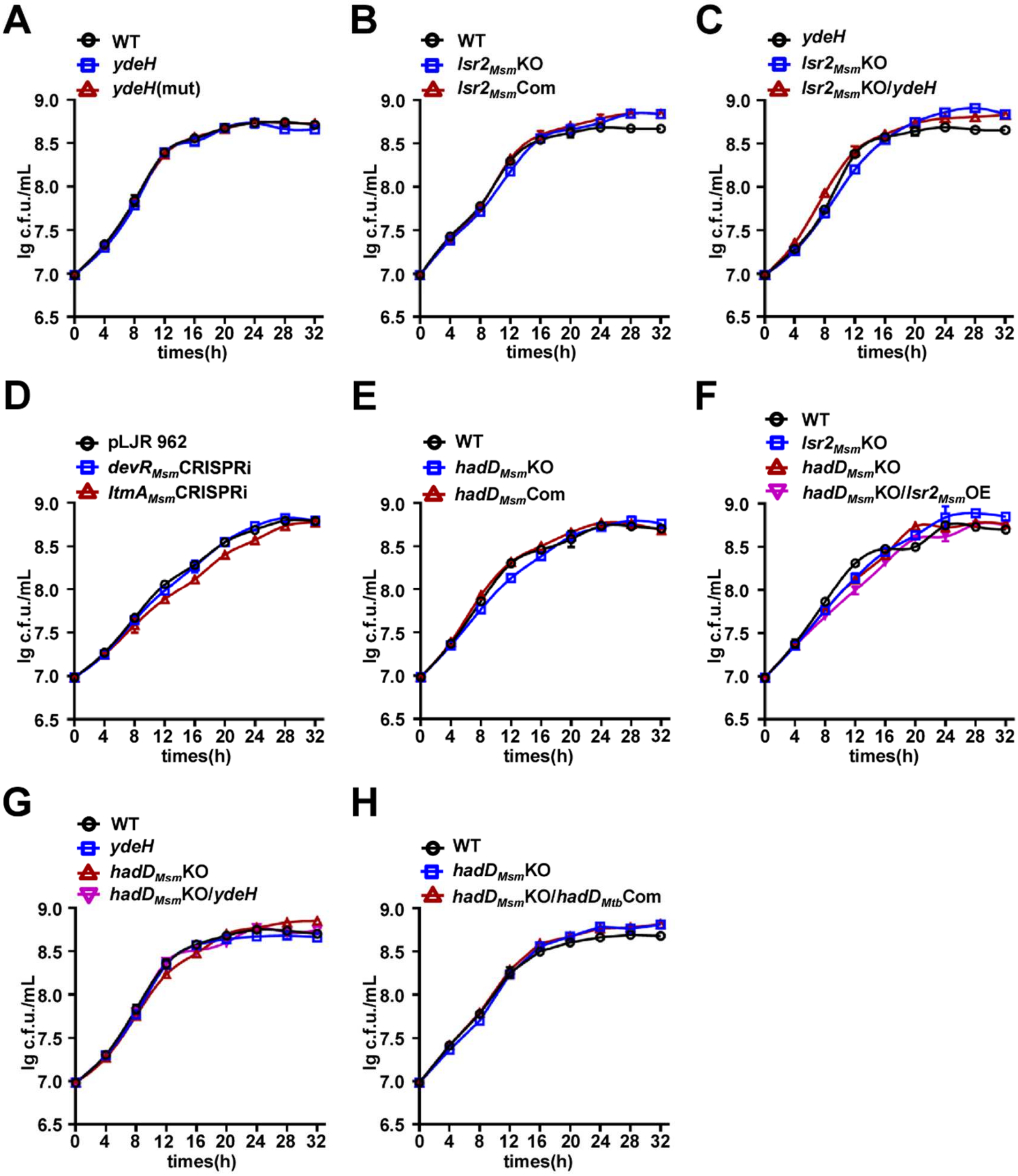

Fig S1. Growth curves for recombinant strains. The detection of growth curves were performed according to the previous procedures with some modifications (PDIM: 23047950). Cultures were obtained and plated on 7H10 medium to determine colony-forming units at the indicated times (*n* = 3, biological replicates). Data were presented as mean ± SD. **A:** Growth curves of the WT, *ydeH* and *ydeH*(mut) strains. **B:** Growth curves of the WT, *lsr2*$_{Msm}$ knock-out and *lsr2*$_{Msm}$ complementary strains. **C:** Growth curves of the *ydeH*, *lsr2*$_{Msm}$ knock-out and *lsr2*$_{Msm}$KO/*ydeH* strains. **D:** Growth curves

of the WT, *hadD*$_{Msm}$ knock-out and *hadD*$_{Msm}$ complementary strains. **E:** Growth curves of the WT, *lsr2*$_{Msm}$ knock-out, *hadD*$_{Msm}$ knock-out and its *lsr2*$_{Msm}$ overexpression strains. **F:** Growth curves of the WT, *ydeH*, *hadD*$_{Msm}$ knock-out and its *ydeH* overexpression strains. **G:** Growth curves of the WT, *hadD*$_{Msm}$ knock-out and its *hadD*$_{Mtb}$ complementary strains. **H:** Growth curves of the Msm/pLJR962, *devR*$_{Msm}$ CRISPRi and *ltmA*$_{Msm}$ CRISPRi strains.

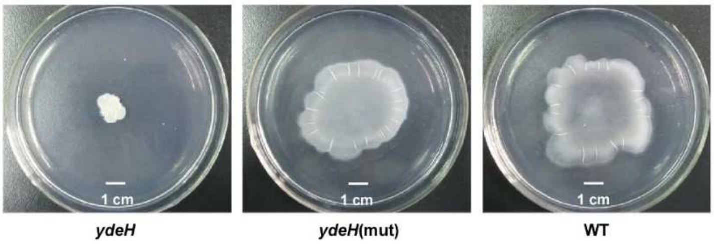

Fig S2. The effect of c-di-GMP on the motility of *M. smegmatis*. The motility of the wide type, *ydeH*(mut) and *ydeH* overexpression strains.

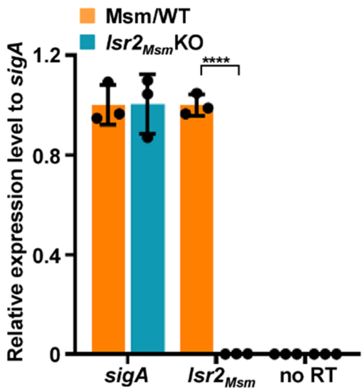

Fig S3. Detection of the expression levels of *lsr2*$_{Msm}$. RT-qPCR assays for determining the relative expression levels of *lsr2*$_{Msm}$ in the Msm/WT and *lsr2*$_{Msm}$KO strains (n = 3, sample replicates). Expression levels of genes were normalized using the *sigA* gene as an invariant transcript. Two-tailed Student's t-tests were performed for statistical analysis of three independent biological experiments (**** $p < 0.0001$). no RT was genomic DNA contamination control. Data were analyzed using the $2^{\Delta\Delta}$Ct method. Data were presented as mean ± SD.

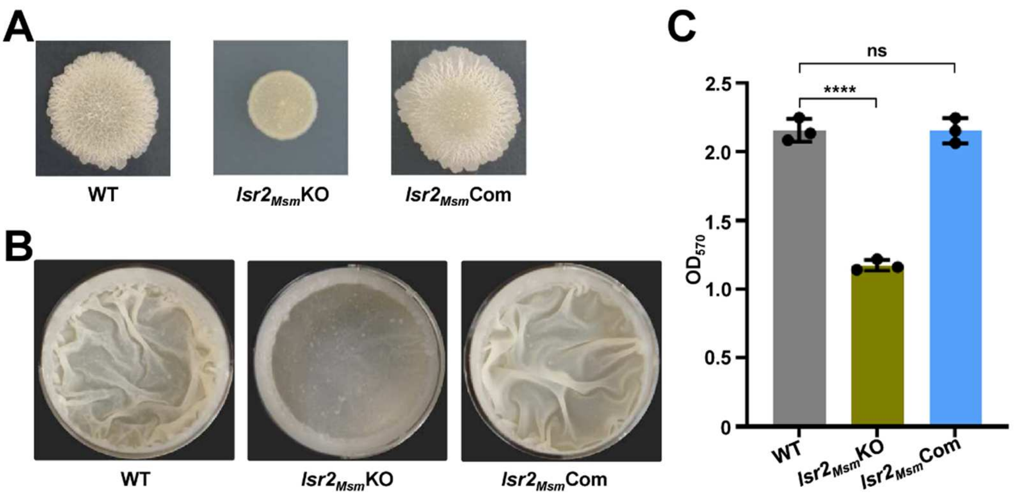

Fig S4. The effect of *lsr2*$_{Msm}$ on colony morphology and biofilm formation of *M. smegmatis*. A: Spot colony morphology of the WT, *lsr2*$_{Msm}$ knock-out and its *lsr2*$_{Msm}$ complementary strains. **B:** Biofilm formation at the air-liquid surface of the WT, *lsr2*$_{Msm}$ knock-out and its *lsr2*$_{Msm}$ complementary strains. **C:** Quantitation of biofilm biomass by crystal violet staining of the wide type, *lsr2*$_{Msm}$ knock-out, and *lsr2*$_{Msm}$-complemented strains Data were presented as mean ± SD (n = 3, biological replicates). Two-tailed t-tests were performed for statistical analysis (****$p < 0.0001$).

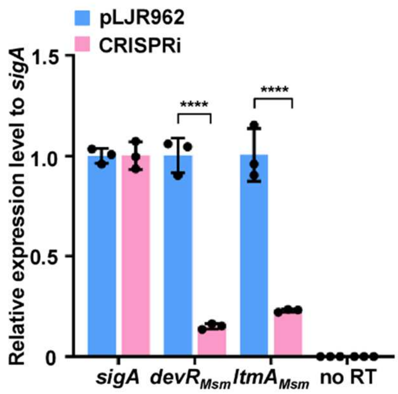

Fig S5. Detection of target genes expression levels in the CRISPRi strains. RT-qPCR assays for determining the relative expression levels of *devR*$_{Msm}$ and *ltmA*$_{Msm}$ in the pLJR962, *devR*$_{Msm}$ CRISPRi or *ltmA*$_{Msm}$ CRISPRi strains (n = 3, sample replicates). Expression levels of genes were normalized using the *sigA* gene as an invariant transcript. Two-tailed Student's t-tests were performed for statistical analysis of three independent biological experiments (****$p < 0.0001$). no RT was genomic DNA contamination control. Data were analyzed using the $2^{\Delta\Delta}$Ct method. Data were presented as mean ± SD.

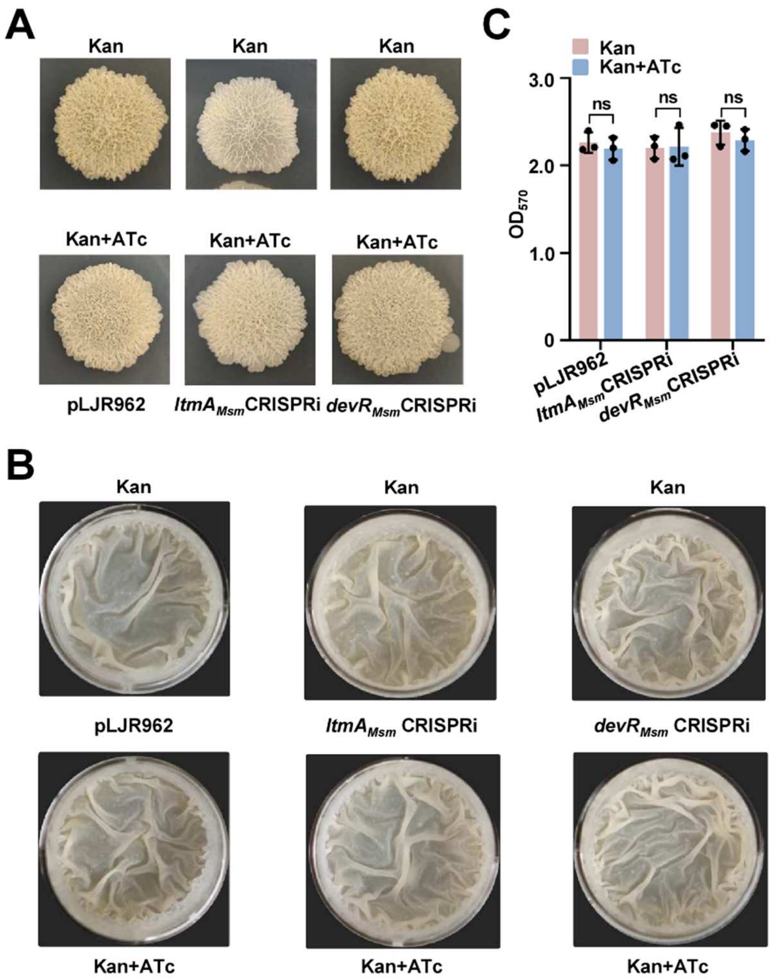

Fig S6. **The effect of *dosR (devR)* and *ltmA* on colony phenotype and biofilm formation of *M. smegmatis*. A:** Spot colony morphology of the *M. smegmatis* strains on 7H10 medium with 30 mg/mL Kan or 30 mg/mL Kan + 200 ng/mL ATc. The strains include pLJR962 (containing empty pLJR962 plasmid), $devR_{Msm}$ CRISPRi (containing pLJR962-$devR_{Msm}$ CRISPRi plasmid) and $ltmA_{Msm}$ CRISPRi (containing $ltmA_{Msm}$ CRISPRi plasmid). **B:** Biofilm formation observation of the pLJR962, $devR_{Msm}$ CRISPRi, and $ltmA_{Msm}$ CRISPRi strains. **C:** Biofilm biomass quantitation of pLJR962, $devR_{Msm}$ CRISPRi, and $ltmA_{Msm}$ CRISPRi strains (n = 3, biological replicates). Two-tailed t-tests were performed for statistical analysis (ns, $p = 0.8269$; ns, $p = 0.3181$; ns, $p = 0.4912$). Data were presented as mean ± SD.

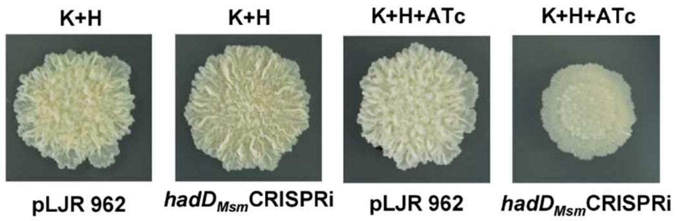

Fig S7. **The effect of $hadD_{Msm}$ and c-di-GMP on colony morphology of *M.***

smegmatis. Spot colony morphology of the high c-di-GMP-content *M. smegmatis* strain that contains an empty pLJR962 plasmid (pLJR962) or pLJR962*hadD*$_{Msm}$ CRISPRi plasmid (*hadD*$_{Msm}$CRISPRi) on 7H10 medium with 30 mg/mL Kan + 50 mg/mL Hyg or 30 mg/mL Kan + 50 mg/mL Hyg + 200 ng /mL ATc.

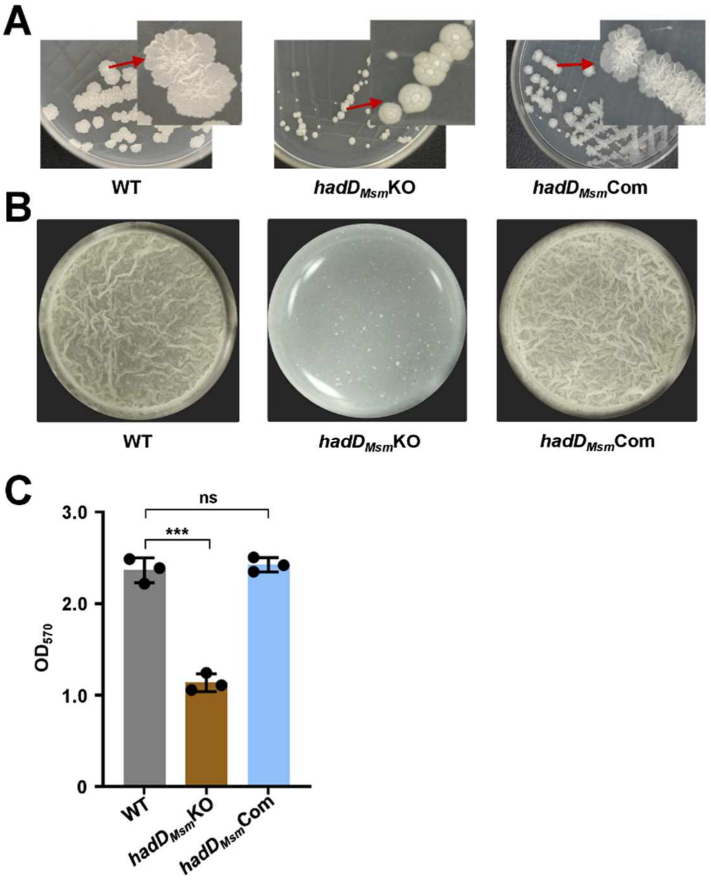

Fig S8. The effect of *hadD*$_{Msm}$ on biofilm formation of *M. smegmatis.* **A**: Colony morphology of the wide type, *hadD*$_{\mathrm{Msm}}$ knock-out, and *hadD*$_{Msm}$-complemented strains. **B**: Biofilm formation of the wide type, *hadD*$_{\mathrm{Msm}}$ knock-out, and *hadD*$_{Msm}$-complemented strains. **C**: Quantitation of biofilm biomass by crystal violet staining of the wide type, *hadD*$_{Msm}$ knock-out, and *hadD*$_{Msm}$-complemented strains. (n = 3, biological replicates). Two-tailed t-tests were performed for statistical analysis (***p = 0.0002; ns, p = 0.5456). Data were presented as mean ± SD.

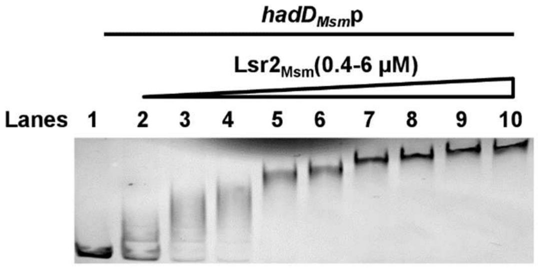

Fig S9. EMSA assays for the DNA-binding activity of Lsr2. The increasing amounts of Lsr2$_{Msm}$ was co-incubated with *hadD$_{Msm}$*p (lanes 2-10). The experiment was performed three times and the representative image was shown.

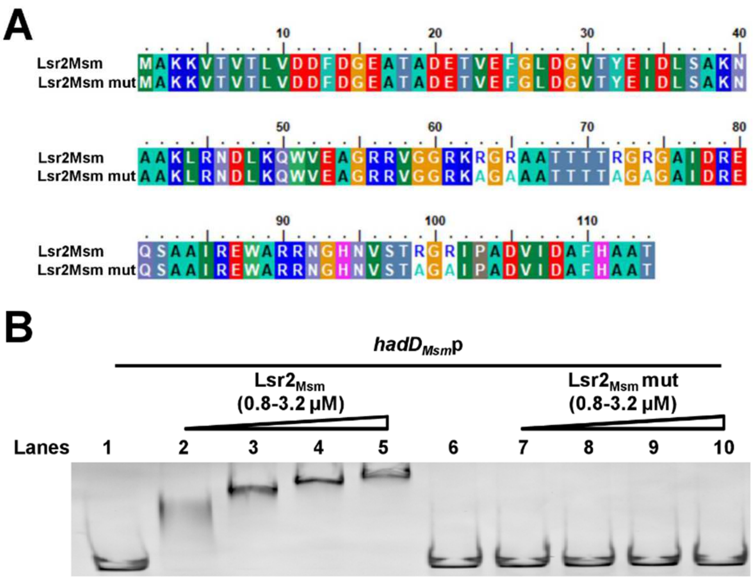

Fig S10. Detection of the DNA-binding activity of Lsr2 mutant protein. A: Amino acid sequence alignment of Lsr2$_{Msm}$ and Lsr2$_{Msm}$ mutant protein. **B:** EMSA assays for the DNA-binding activity of Lsr2$_{Msm}$ and Lsr2$_{Msm}$ mutant protein. *hadD$_{Msm}$*p was co-incubated with increasing amounts of Lsr2$_{Msm}$ (lanes 2-5) and Lsr2$_{Msm}$ mutant protein (lanes 7-10). The experiment was performed three times and the representative image was shown.

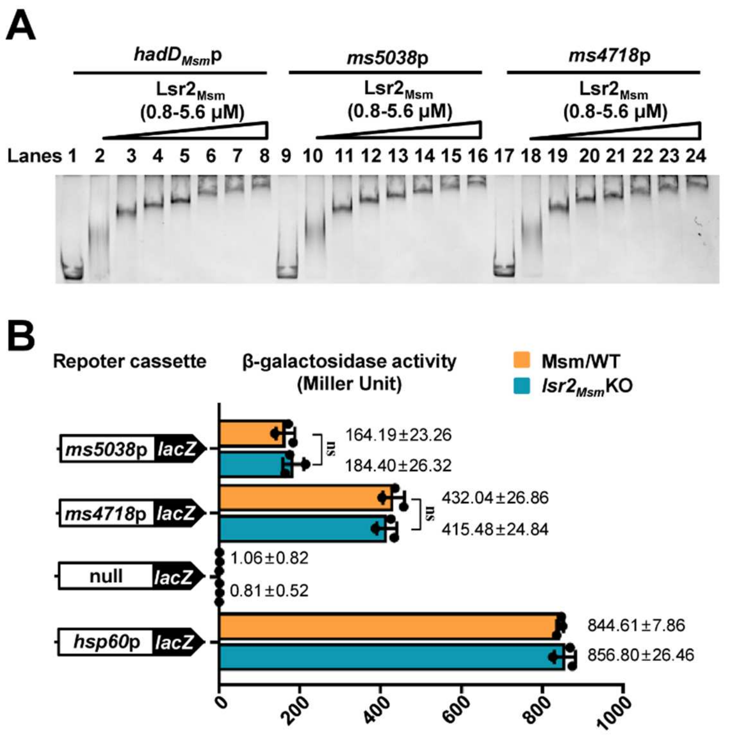

Fig S11. Detection the specific regulation of Lsr2. A: EMSA assays for the DNA-binding activity of Lsr2. The increasing amounts of Lsr2 was co-incubated with *hadD*$_{Msm}$p (lanes 1-8), *ms5038*p (lanes 9-16) and *ms4718*p (lanes 17-24). The experiment was performed three times and the representative image was shown. **B:** The β-galactosidase activity experiment to assay for the effect of Lsr2 on the expression of *ms5038* and *ms4718* (n = 3, biological replicates). None promoter-*lacZ* and *hsp60*p-*lacZ* were used as controls. The data were presented as Miller units on the right panel. Left column: schematic representation of each plasmid used to construct recombinant strains. Two-tailed Student's t-tests were performed for statistical analysis of three independent biological experiments (ns, p = 0.7627; ns, p = 0.8055). Data were presented as mean ± SD.

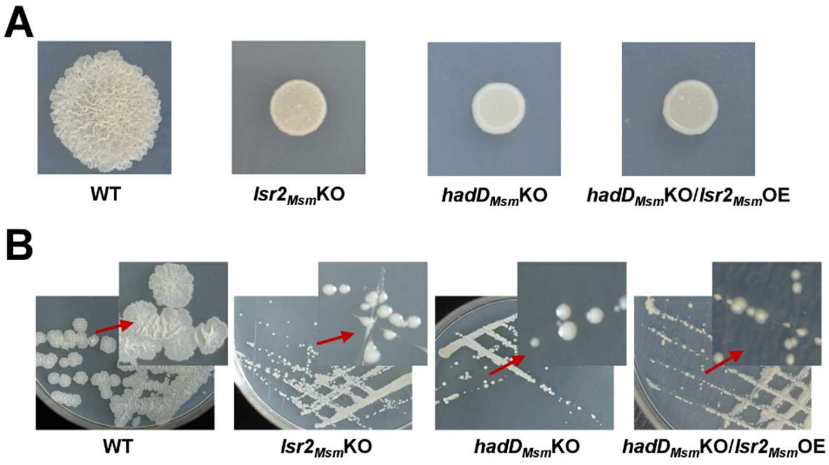

Fig S12. The effect of *lsr2*$_{Msm}$ and *hadD*$_{Msm}$ on biofilm formation of *M. smegmatis*. **A**: Spot colony morphology of the wide type, *lsr2*$_{Msm}$ knock-out, *hadD*$_{Msm}$ knock-out and its *lsr2*$_{Msm}$-overexpressed strains. **B**: Colony morphology of the wide type, *lsr2*$_{Msm}$ knock-out, *hadD*$_{Msm}$ knock-out, and *lsr2*$_{Msm}$-overexpressed strains.

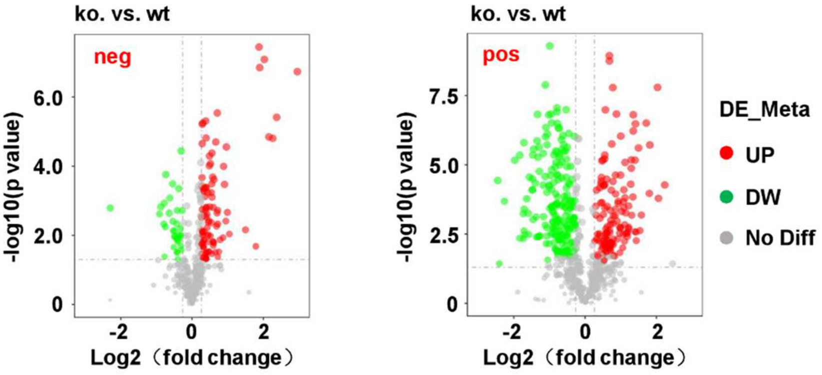

Fig S13. Volcano plot analysis of the lipid. The difference of the lipid between the WT and *lsr2*$_{Msm}$KO *M. smegmatis* strains was detected by lipidomic assays in negative ion mode (right) and positive ion mode (left). The horizontal coordinate indicates the change of content ratio of lipid compounds in different groups (log2 (fold change)), and the vertical coordinate indicates the significance level of difference (-log10 (*p* value)). The significantly upregulated lipid compounds were represented by red dots, and the significantly down-regulated lipid compounds were indicated by green dots.

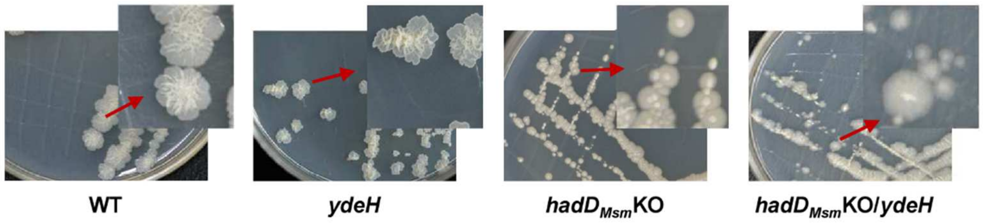

Fig S14. The effect of *hadD*$_{Msm}$ and c-di-GMP on colony phenotype of *M. smegmatis*. Colony morphology of the wide type and *ydeH*-overexpressed, *hadD*$_{Msm}$ knock-out and its *ydeH*-overexpressed strains.

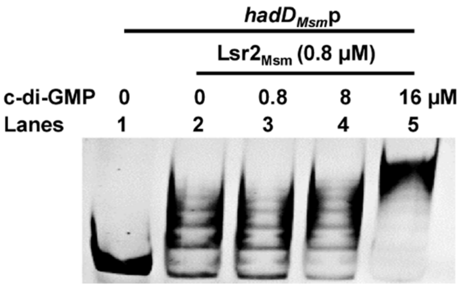

Fig S15. Detection for the effect of c-di-GMP on DNA-binding activity of Lsr2.

EMSA assays for the effect of c-di-GMP on the DNA-binding activity of $Lsr2_{Msm}$. The increasing amounts of c-di-GMP (0.8-16 μM) (lanes 3-5) were added to the reactions and incubated with $Lsr2_{Msm}$ for 15 min. The experiment was performed three times and the representative image was shown.

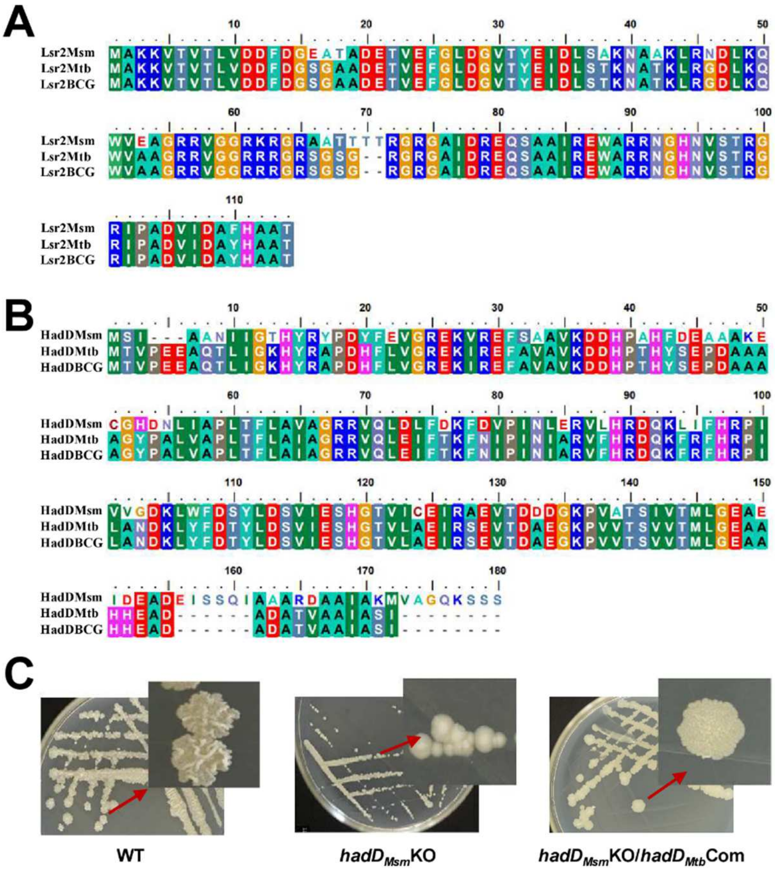

Fig S16. The function of Lsr2 and HadD on colony morphology is conservative in mycobacteria. A: Amino acid sequence alignment of Lsr2 between *M. smegmatis*, *M. tuberculosis,* and *M. bovis* BCG. **B**: Amino acid sequence alignment of HadD between *M. smegmatis*, *M. tuberculosis*, and *M. bovis* BCG. **C**: Colony morphology of the wide type, *$hadD_{Msm}$* knock-out and *$hadD_{Mtb}$*-complemented strains.

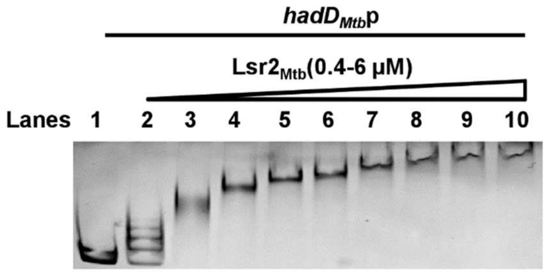

Fig S17. EMSA assays for the DNA-binding activity of Lsr2. The increasing amounts of $Lsr2_{Mtb}$ was co-incubated with *$hadD_{Mtb}$*p (lanes 2-10). The experiment was

performed three times and the representative image was shown.

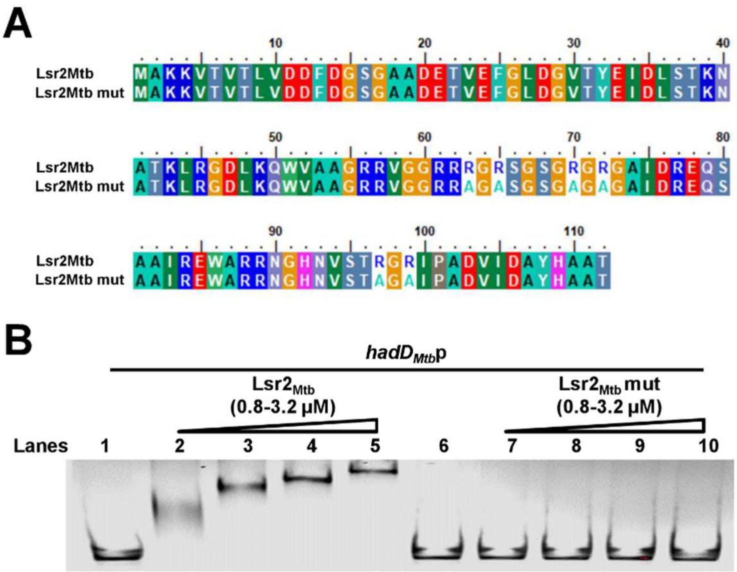

Fig S18. Detection for the DNA-binding activity of Lsr2 mutant protein. A: Amino acid sequence alignment of Lsr2$_{Mtb}$ and Lsr2$_{Mtb}$ mutant protein. **B:** EMSA assays for the DNA-binding activity of Lsr2$_{Mtb}$ and Lsr2$_{Mtb}$ mutant protein. *hadD$_{Mtb}$*p was co-incubated with increasing amounts of Lsr2$_{Mtb}$ (lanes 2-5) and Lsr2$_{Mtb}$ mutant protein (lanes 7-10). The experiment was performed three times and the representative image was shown.

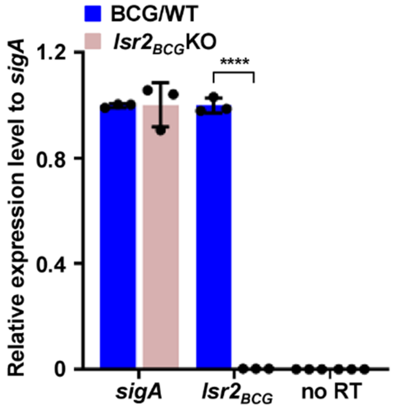

Fig S19. Detection for the expression levels of *lsr2$_{BCG}$*. RT-qPCR assays for determining the relative expression levels of *lsr2$_{BCG}$* in the BCG/WT and *lsr2$_{BCG}$*KO strains. Expression levels of genes were normalized using the *sigA* gene as an invariant transcript (n = 3, sample replicates). Two-tailed Student's t-tests were performed for statistical analysis of three independent biological experiments (**** $p < 0.0001$). no RT was genomic DNA contamination control. Data were analyzed using the $2^{\Delta\Delta}$Ct method. Data were presented as mean ± SD.

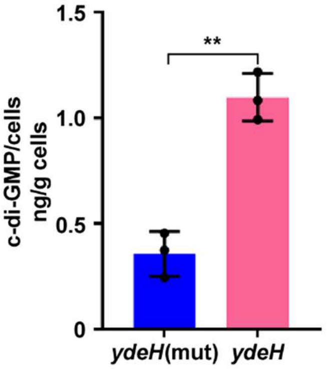

Fig S20. Detection of the levels of c-di-GMP in *M. bovis* BCG. The *yedH*(mut)-overexpression (*yedH*(mut)) and *yedH*-overexpression (*yedH*) of *M. bovis* BCG were constructed for detecting the levels of c-di-GMP. Data were presented as mean ± SD (*n* = 3, biological replicates). The c-di-GMP of *M. bovis* BCG strains were extracted using a modified protocol according to the previously procedures (PMID: 23047950). The strains were cultured to an OD_{600}=1.2, and cells were collected and washed twice with PBS buffer. Subsequently, cells were suspended in 15 mL ddH_2O and crushed using ultrasound at 450 W for 1h. The samples were centrifuged. Then, the supernatant was extracted with phenol/chloroform and concentrated to 1.5 mL. Finally, the levels of c-di-GMP were quantified using an ELISA kit (FANKEWEI, China). Then, the intracellular levels of c-di-GMP were converted to nanogram per gram (wet weight cells). Two- tails t-tests were performed for statistical analysis (***p* = 0.0012).

Primer list (Supplementary Table S1)

Table S1. The primers used in this study

Name	Sequence 5' to 3'	General description
*Eco*R I *ydeH* f	ACGC GAATTC ATGATCAAGAAGACAACG	*ydeH*, *ydeH*(mut)
Xba I *ydeH* r	ACGC TCTAGA TTAAACTCGGTTAATCAC	overexpression
LAD2	AGTCTCGCAGATGATAAGGBNBNNNGGTT	Transposon insertion
Trans-f	CGCTTCCTCGTGCTTTACGGTATCG	mutant sequence
Trans-r	CCCGAAAAGTGCCACCTAAATTGTAAGCG	analysis
Adapter	AGTCTCGCAGATGATAAGG	
Pac I *lsr2*$_{Msm}$up f	TATA TTAATTAA CTCGCCGGCGGTGCCGCCGC	*lsr2*$_{Msm}$ gene knockout
Spe I *lsr2*$_{Msm}$up r	TATA ACTAGT CACTCCCGGGCACCGACCCTTCT	
Hind III *lsr2*$_{Msm}$dn f	TATA AAGCTT CGGAAGGTTGCCAGCACGCC	
Nhe I *lsr2*$_{Msm}$udn r	ATAT GCTAGC GTCCTTGAGCGTCTCGGGGA	
Pac I *lsr2*$_{BCG}$up f	GCATTTAATTAATTAGCCGGTGGTGCGGCGGC	*lsr2*$_{BCG}$ gene knockout
Spe I *lsr2*$_{BCG}$up r	AGCGACTAGTCACTGGCTTACCCTCGCGTTTCT	
*Hin*d III *lsr2*$_{BCG}$dn f	AGGCAAGCTTCATTTGGCTACCGGCGCCCA	
Nhe I *lsr2*$_{BCG}$dn r	ATTAGCTAGCGCGTCTCGGGCACCTCGCCG	

Hyg f	AGCCAGCGCATATGGTGACACAAGAATCCCTG	Gene knockout strain
Hyg r	ACACTTAATTAATTAGGCGCCGGGGGCGGT	*hyg* analysis
Not I $lsr2_{Msm}$ f	AGAAT GCGGCCGC T ATGGCAAAGAAAGTGAC	$Lsr2_{Msm}$ protein
Xba I $lsr2_{Msm}$ r	ATGC TCTAGA CTAAGTTGCCGCGTGGAATG	expression
Not I $lsr2_{BCG}$ f	AGAAT GCGGCCGC T ATGGCGAAGAAAGTAAC	$Lsr2_{BCG}$ protein
Xba I $lsr2_{BCG}$ r	ATGC TCTAGATCAGGTCGCCGCGTGGTATG	expression
$hadD_{Msm}$ sgRNA f	GGGAACTCGACGGCCCGCGACCGC	$hadD_{Msm}$ CRISPRi
$hadD_{Msm}$ sgRNA r	AAACGCGGTCGCGGGCCGTCGAGT	plasmid
CRI test f	TTACGCTGACTTGACGGGACGGC	Gene CRISPRi strain
CRI test r	GTTCCTGGCCTTTTGCTGGC	sequence analysis
$hadD_{Msm}$ up f	GCATTTAATTAA GCCGGGGCGTCAGAACGGGC	
$hadD_{Msm}$ up r	AGCGACTAGT CACGTGTTCTCTGCTACTCCCGT	$hadD_{Msm}$
$hadD_{Msm}$ dn f	ATTAAAGCTT GTTCACCCGCCGGGCGGAGG	gene knockout
$hadD_{Msm}$ dn r	ATTAGCTAGC ACGCAGGCATCCGTCGCTGC	
*Eco*R I $hadD_{Msm}$ f	AGCGGAATTCATGAGCATCGCGGCAAACAT	$hadD_{Msm}$ gene
Xba I $hadD_{Msm}$ r	ACCGTCTAGATCAGGAACTGGACTTTTGCC	overexpression
*Eco*R I $hadD_{Msm}$p f	ATTAGAATTC GACGCCGCGCTGCGCGACGT	The β-galactosidase
Xba I $hadD_{Msm}$p r	ATTATCTAGA TAGTCGGGGTAGCGGTAGTG	activity experiment
$hadD_{Msm}$p f	GACGCCGCGCTGCGCGACGT	EMSA for the hadDp
$hadD_{Msm}$p r	TAGTCGGGGTAGCGGTAGTG	binding activity of
$hadD_{Mtb}$p f	CCGCTCGGATCGGGGTTC	Lsr2
$hadD_{Mtb}$p r	GCCCGATAATGCTTGCCGAT	
RT$hadD_{Msm}$ f	AGAAGGTCCGCGAGTTCTC	RT-PCR analysis for
RT$hadD_{Msm}$ r	ATCGGACGGTGGAAGATCAG	the expression of
$sigA_{Msm}$ f	GCATCGGCCGACTCGGTTCG	$hadD_{Msm}$
$sigA_{Msm}$ r	GCGACACCACGAGGCGCAGG	
Kpn I *his* f	ATTCGGTACCATGGGCAGCAGCCATCATCA	ChIP assay for c-di-
Not I $hadD_{Msm}$ r	AGAATGCGGCCGCCTAAGTTGCCGCGTGGAATG	GMP modulates the
RT $hadD_{BCG}$p f	AGCCTCTAGACGCCAATCAC	hadDp-binding
RT $hadD_{BCG}$p r	GCTTCTTCGGGAACTGTCAT	activity of Lsr2
*Bma*H I *ydeH* f	ACGC GGATCC ATGATCAAGAAGACAACG	
TF$hadD_{Msm}$ r	TCCGTTGTCTTCTTGATCATCCTTGACGGCCGCC GAGAA	The β-galactosidase activity experiment
$hadD_{Msm}$ *ydeH* f	TTCTCGGCGGCCGTCAAGGATGATCAAGAAGAC AACGGA	for c-di-GMP modulates the *hadD*p-
Sal I *ydeH* r	GCGCGTCGACTTAAACTCGGTTAATCAC	binding activity of
Not I $lsr2_{BCG}$ f	AGAATGCGGCCGCTCAGGTCGCCGCGTGGTATG	Lsr2
*Eco*R I $hadD_{Mtb}$ f	ATCTGAATTCATGACAGTTCCCGAAGAAGC	
Xba I $hadD_{Mtb}$ r	ACCGTCTAGACTAGATCGATGCAATCGCCG	
RT $hadD_{BCG}$ f	GATCGGCAAGCATTATCGGG	RT-PCR analysis for
RT $hadD_{BCG}$ r	TTCTGGTCGCGATGAAAGAC	the expression of
$sigA_{BCG}$ f	TCGCGCCTACCTCAAACAG	$hadD_{BCG}$
$sigA_{BCG}$ r	CGTACAGGCCAGCCTCGAT	
