# Supplementary material for: Lsr2 acts as a cyclic di-GMP receptor that promotes keto-mycolic acid synthesis and biofilm formation in mycobacteria
Source: Nat Commun. 2024 Jan 24;15:695. doi: 10.1038/s41467-024-44774-6 (PMC10808224; doi:10.1038/s41467-024-44774-6)
Supplement: Supplementary file 2 — Reporting Summary [file 41467_2024_44774_MOESM2_ESM.pdf]

nature portfolio

Corresponding author(s): Weihui, Li

Last updated by author(s): Dec 17, 2023

Reporting Summary

Nature Portfolio wishes to improve the reproducibility of the work that we publish. This form provides structure for consistency and transparency in reporting. For further information on Nature Portfolio policies, see our Editorial Policies and the Editorial Policy Checklist.

Statistics

For all statistical analyses, confirm that the following items are present in the figure legend, table legend, main text, or Methods section.

n/a	Confirmed	
☐	☒	The exact sample size (*n*) for each experimental group/condition, given as a discrete number and unit of measurement
☐	☒	A statement on whether measurements were taken from distinct samples or whether the same sample was measured repeatedly
☐	☒	The statistical test(s) used AND whether they are one- or two-sided<br>*Only common tests should be described solely by name; describe more complex techniques in the Methods section.*
☒	☐	A description of all covariates tested
☐	☒	A description of any assumptions or corrections, such as tests of normality and adjustment for multiple comparisons
☐	☒	A full description of the statistical parameters including central tendency (e.g. means) or other basic estimates (e.g. regression coefficient) AND variation (e.g. standard deviation) or associated estimates of uncertainty (e.g. confidence intervals)
☐	☒	For null hypothesis testing, the test statistic (e.g. *F*, *t*, *r*) with confidence intervals, effect sizes, degrees of freedom and *P* value noted<br>*Give P values as exact values whenever suitable.*
☒	☐	For Bayesian analysis, information on the choice of priors and Markov chain Monte Carlo settings
☒	☐	For hierarchical and complex designs, identification of the appropriate level for tests and full reporting of outcomes
☒	☐	Estimates of effect sizes (e.g. Cohen's *d*, Pearson's *r*), indicating how they were calculated

Our web collection on statistics for biologists contains articles on many of the points above.

Software and code

Policy information about availability of computer code

Data collection	ITC assays were carried out with a Nano ITC Low Volume isothermal calorimeter (TA Instruments, New Castle, DE, USA); Micrographs of mycobacteria were observed by Quattro scanning electron microscopy (OPTON, China) and OLYMPUS CX33 optical microscope (Olympus Corporation, Japan); Images of gels were acquired by Geldoc scanner (Bio-Rad, USA); RNA and protein quantification were performed through NanoDrop OneC (Thermo Fisher, Germany); RT-qPCR was performed by QuantStudio 3 Real-Time PCR System (Thermo Fisher, Germany); Biofilm quantitation, the β-galactosidase activity experiment and the detection of c-di-GMP concentration were measured using a TECAN Infinite M200 Pro Nano Quant microplate reader (Mannedorf, Switzerland). The lipids were detected by UHPLC-MS/MS using a Vanquish UHPLC system (Thermo Fisher, Germany) coupled with an Orbitrap Q ExactiveTM HF mass spectrometer (Thermo Fisher, Germany).
Data analysis	The data of ITC were analyzed using the NanoAnalyze Software (v.3.11.0); The statistical analysis were performed using Graphpad Prism (v.8.3.0.538), and two-tailed Student's t-tests or two-tailed t-tests were performed for statistical analysis. The Compound Discoverer 3.01 (CD3.1, Thermo Fisher) was used to perform peak alignment, peak picking, and quantitation of lipidomic raw data. Statistical analyses were performed using the statistical software R (R version R-3.4.3), Python (Python 2.7.6 version) and CentOS (CentOS release 6.6).

For manuscripts utilizing custom algorithms or software that are central to the research but not yet described in published literature, software must be made available to editors and reviewers. We strongly encourage code deposition in a community repository (e.g. GitHub). See the Nature Portfolio guidelines for submitting code & software for further information.

Data

Policy information about availability of data

All manuscripts must include a data availability statement. This statement should provide the following information, where applicable:

- Accession codes, unique identifiers, or web links for publicly available datasets
- A description of any restrictions on data availability
- For clinical datasets or third party data, please ensure that the statement adheres to our policy

The mass spectrometry of lipidomic data generated in this study have been deposited in the MetaboLights database [www.ebi.ac.uk/metabolights/MTBLS7053]. The processed lipidomic data are available at Source Data file. Mtb LipidDB (https://www.ncbi.nlm.nih.gov/pmc/articles/PMC3073466/) was used for metabolite identification of lipidomic. The absorbance, gels and EMSA data generated in this study were provided in Source Data file.

Research involving human participants, their data, or biological material

Policy information about studies with human participants or human data. See also policy information about sex, gender (identity/presentation), and sexual orientation and race, ethnicity and racism.

Reporting on sex and gender	Our study did not involve humans, vertebrates, or cell lines.
Reporting on race, ethnicity, or other socially relevant groupings	Our study did not involve race, ethnicity, or other socially relevant groupings.
Population characteristics	Not applicable.
Recruitment	Not applicable.
Ethics oversight	Not applicable.

Note that full information on the approval of the study protocol must also be provided in the manuscript.

Field-specific reporting

Please select the one below that is the best fit for your research. If you are not sure, read the appropriate sections before making your selection.

☒ Life sciences ☐ Behavioural & social sciences ☐ Ecological, evolutionary & environmental sciences

For a reference copy of the document with all sections, see nature.com/documents/nr-reporting-summary-flat.pdf

Life sciences study design

All studies must disclose on these points even when the disclosure is negative.

Sample size	Sample size for morphological and biofilm observation, biofilm biomass, RT-PCR, chromatin immunoprecipitation assay, ITC assay, β-galactosidase activity assays, and c-di-GMP measurement in M. smegmatis, and M. bovis BCG strains or in vitro were conducted according to our laboratory experience manual and previous research (PMID: 35315431). Comparative lipidomic analysis was performed at Novo gene (Beijing, China).
Data exclusions	no data exclusions
Replication	Three independent biological replicates were performed for biofilms quantitation, β-galactosidase activity experiment, c-di-GMP concentration detection experiment, RT-PCR, EMSA and micrographs observed of mycobacteria. Six independent biological replicates were performed in lipidomic, and five independent biological replicates were used for significance analysis and showed in the figure.
Randomization	No samples were allocated into experimental groups.
Blinding	No samples were allocated into experimental groups.

Reporting for specific materials, systems and methods

We require information from authors about some types of materials, experimental systems and methods used in many studies. Here, indicate whether each material, system or method listed is relevant to your study. If you are not sure if a list item applies to your research, read the appropriate section before selecting a response.

Materials & experimental systems

n/a	Involved in the study	
☐	☒	Antibodies
☒	☐	Eukaryotic cell lines
☒	☐	Palaeontology and archaeology
☒	☐	Animals and other organisms
☒	☐	Clinical data
☒	☐	Dual use research of concern
☒	☐	Plants

Methods

n/a	Involved in the study	
☒	☐	ChIP-seq
☒	☐	Flow cytometry
☒	☐	MRI-based neuroimaging

Antibodies

Antibodies used	6*His Monoclonal Antibody was produced in Mouse by CUSABIO (China), catalog number: CSB-MA000011M0m , clone name: 3G5H8 , and lot number: J0908.
Validation	The antibody was verified by Western Blot experiment. Positive WB detected in: His fusion protein at 20 ng, 10 ng, 5 ng, 2.5 ng with 6*His antibody at 1:1000. The antibody was verified by application in the research:<br>Dominguez G.et al. Neuronal sphingosine kinase 2 subcellular localization is altered in Alzheimer's disease brain. Acta Neuropathol Commun, 2018, 3;6(1):25.
